# Rare Peritoneal Involvement in Adult T-Cell Leukemia/Lymphoma: A Diagnostic Conundrum

**DOI:** 10.7759/cureus.87342

**Published:** 2025-07-05

**Authors:** Nithya Krishnamurthy, Bruce Petersen, Christian Farag, Anthony Hafez, Raksha Kulkarni, Busra Cangut, Roshan Asrani

**Affiliations:** 1 Department of Internal Medicine, Icahn School of Medicine at Mount Sinai, New York, USA; 2 Department of Pathology, Icahn School of Medicine at Mount Sinai, New York, USA; 3 Department of Nuclear Medicine, Icahn School of Medicine at Mount Sinai, New York, USA; 4 Department of Nuclear Medicine, Mount Sinai Hospital, New York, USA; 5 Department of Oncology, Icahn School of Medicine at Mount Sinai, New York, USA

**Keywords:** adult t-cell leukemia-lymphoma (atll), malignant ascites, peritoneal malignancy, pet-ct, pet-ct with 18f-fdg

## Abstract

We present the case of a 60-year-old Caribbean man with no significant past medical history who presented to the emergency department with a three-week history of progressive, diffuse abdominal pain associated with early satiety and unintentional weight loss. Vital signs were stable, and physical examination revealed abdominal distension and diffuse tenderness without peritoneal signs. Laboratory studies were notable for elevated lactate dehydrogenase and mild leukocytosis. Computed tomography followed by PET-CT imaging demonstrated multiple nodular soft tissue densities with increased fluorine-18 fluorodeoxyglucose (18F-FDG) uptake throughout the mesentery, peritoneum, and bilateral pleural surfaces, raising concern for peritoneal lymphomatosis.

An ultrasound-guided biopsy of an omental implant was performed, revealing large atypical lymphoid cells positive for CD3, CD2, and CD4, and negative for CD5, CD7, and CD8, with a Ki-67 proliferation index of 90%, consistent with an aggressive peripheral T-cell lymphoma. Peripheral blood RT-PCR returned positive for human T-cell lymphotropic virus type 1 (HTLV-1), aligning with the diagnosis of acute adult T-cell leukemia/lymphoma (ATLL), stage IV.

The patient’s clinical course was rapidly progressive and marked by complications including spontaneous tumor lysis syndrome, recurrent episodes of malignant small bowel obstruction, and gram-negative bacteremia. While preparing for treatment with dose-adjusted etoposide phosphate, vincristine sulfate (Oncovin), cyclophosphamide, and doxorubicin hydrochloride (hydroxydaunomycin) (EPOCH) chemotherapy, he suffered a cardiac arrest during central venous catheter placement. Despite resuscitation efforts, he sustained an anoxic brain injury and died 32 days after the initial presentation.

This case highlights an aggressive presentation of ATLL with peritoneal involvement and underscores the challenges of rapid disease progression and acute complications, as well as the importance of considering endemic exposure history, utilizing PET-CT for identifying extranodal disease, and rapidly confirming diagnosis through biopsy.

## Introduction

Peritoneal lymphomatosis (PL) refers to the spread of lymphoma cells within the peritoneum. While PL is occasionally observed in advanced stages of lymphoma, it is rare, accounting for approximately 0.75% of all lymphoma cases, and is predominantly associated with high-grade B-cell lymphoma [1,2]. Radiologically, PL closely mimics peritoneal carcinomatosis (PC), making an accurate diagnosis challenging without histopathological confirmation [3]. Here, we present a case of peritoneal involvement in adult T-cell leukemia/lymphoma (ATLL), a rare and aggressive subtype of T-cell lymphoma, and emphasize the importance of diagnostic modalities in identifying an uncommon clinical presentation of this rare disease.

This article was previously presented as a meeting poster at the 2025 Society of General Internal Medicine (SGIM) Annual Scientific Meeting on May 15, 2025.

## Case presentation

We present a case of a 60-year-old Caribbean man with no significant past medical history who presented to the emergency department with a three-week history of abdominal pain. A computed tomography (CT) scan of the abdomen and pelvis revealed findings highly suggestive of peritoneal carcinomatosis with extensive omental nodularity and large-volume ascites.

Clinical course before admission

Three weeks before admission, the patient presented to an outside hospital with abdominal swelling. An abdominal CT scan revealed diffuse nodularity throughout the omentum and significant abdominopelvic ascites. Over three subsequent visits to outside hospitals, he underwent a diagnostic paracentesis, which demonstrated a serum-ascites albumin gradient (SAAG) of 0, with 4% polymorphonuclear leukocytes, and an ascitic lactate dehydrogenase (LDH) level of 1,372 U/L, collectively indicating the presence of an exudate. Cytologic analysis of the ascitic fluid was not conducted and is therefore unavailable. The patient was discharged with spironolactone and instructions to follow up with a liver clinic.

**Figure 1:**
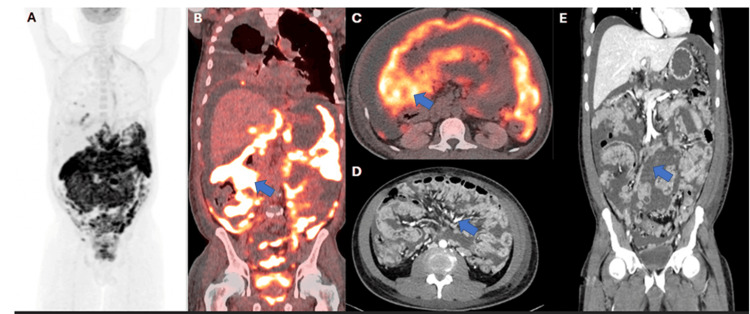
PET/CT imaging (A) Maximum intensity projection (MIP) 18F-FDG PET image. ​(B, C) Coronal and axial planes of PET/CT images demonstrate extensive FDG uptake throughout the peritoneum and mesentery, consistent with peritoneal lymphomatosis (see arrows).​ (D, E) Contrast-enhanced CT of the abdomen and pelvis from 10 days prior to the PET/CT, in axial and coronal planes, demonstrates large-volume ascites with diffuse peritoneal and omental implants (see arrows).​ 18F-FDG: fluorine-18 fluorodeoxyglucose

He returned to the emergency department a week later due to worsening symptoms, including new-onset scrotal and penile edema. Repeat CT of the abdomen and pelvis again showed extensive peritoneal nodularity and large-volume ascites. A PET/CT demonstrated nodules with increased fluorine-18 fluorodeoxyglucose (18F-FDG) uptake throughout the mesentery, peritoneum, and bilateral lower lung pleura (Figure 1).

Diagnostic workup

A repeat paracentesis with cytological analysis revealed atypical lymphocytes. The patient's laboratory findings are summarized in Table 1. CT imaging of the chest showed no evidence of a primary lung malignancy. Due to the inability to tolerate bowel preparation, colonoscopy and esophagogastroduodenoscopy (EGD) were deferred.

**Table 1:** Laboratory test results The first three lab tests were conducted before admission.

Lab test	Result	Normal range
Serum-ascites albumin gradient (SAAG) (pre-admission)	0	> 1.1 (exudate if ≤1)
Polymorphonuclear leukocytes (PMNs) (pre-admission)	4%	0.05%
Ascitic lactate dehydrogenase (LDH) (pre-admission)	1,372 U/L	125–220 U/L
White blood cell count (WBC)	11,700/uL	1,000–4,800/uL
Monocytes	1,800/uL	0–800/uL
Neutrophils	8,900/uL	1,500–8,000/uL
Lactate dehydrogenase (LDH)	853 U/L	100–250 U/L
Calcium (adjusted)	10.5 mg/dL	8.5–10.2 mg/dL
Alpha-fetoprotein (AFP)	15.3 ng/mL	<10 ng/mL
Carcinoembryonic antigen (CEA)	Normal	<10 ng/mL
Cancer antigen 19–9 (CA 19–9)	Normal	<37 U/mL
Beta-human chorionic gonadotropin (β-hCG)	Normal	<5 IU/L

**Figure 2:**
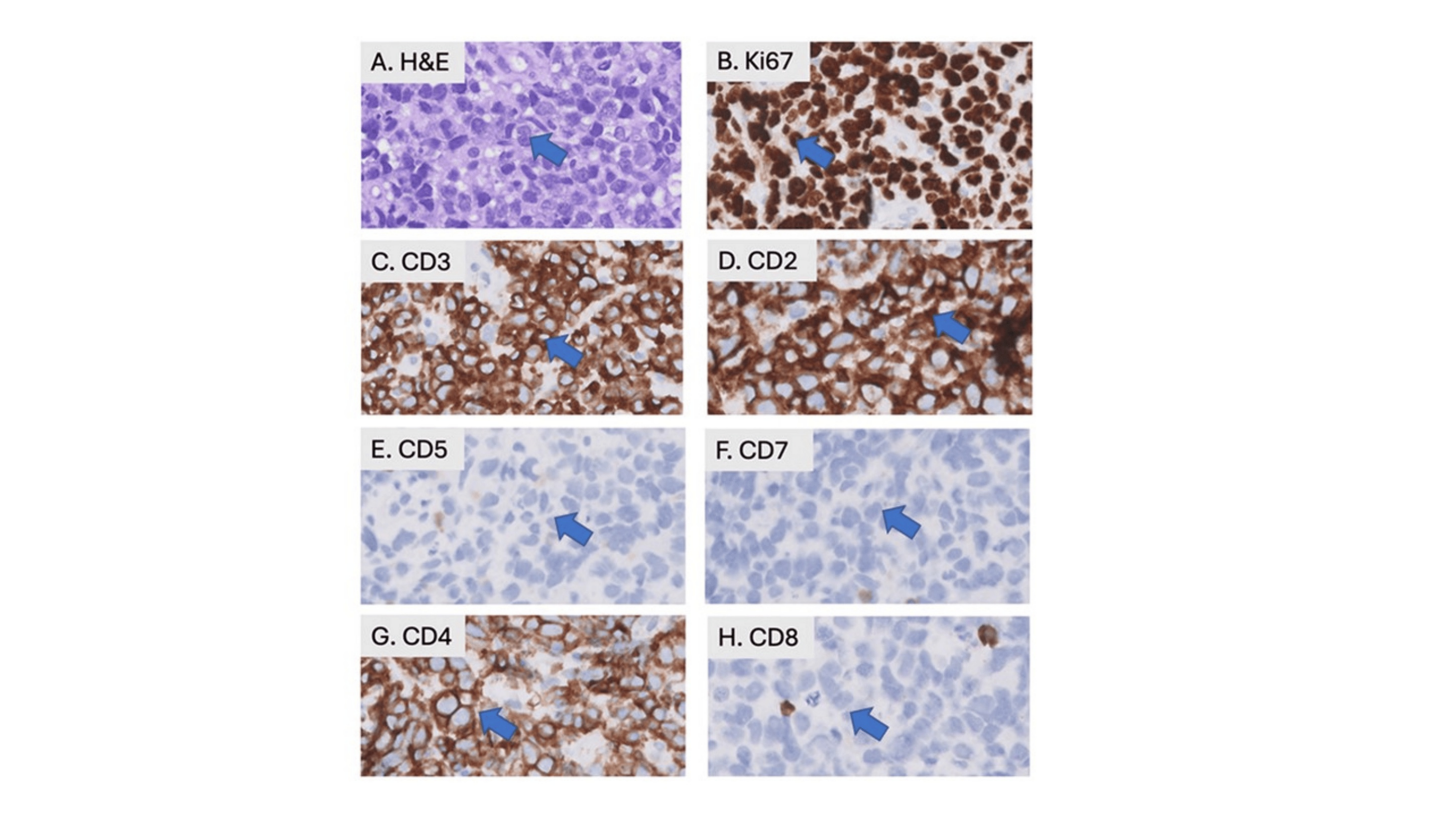
Immunohistochemistry suggesting aggressive T-cell lymphoma (A) H&E stain of left upper quadrant omental mass biopsy showing confluent sheets of large atypical lymphoid cells (see arrow). (B-H) The atypical cells show a proliferation index of approximately 90% by labeling for Ki67, are positive for T-lineage markers (CD3, CD2), show loss of CD5, CD7, and are uniformly positive for CD4, while negative for CD8 (see arrows) (original magnifications X400). H&E: hematoxylin and eosin

Ultrasound-guided biopsy of an omental implant for confirmation revealed large atypical lymphoid cells showing an aberrant T-lineage phenotype by immunohistochemistry (CD3+, CD2+, CD4+, CD8-, CD5-, CD7-) with Ki67 proliferative index of approximately 90%, indicative of an aggressive T-cell lymphoma (Figure 2). Peripheral blood real-time polymerase chain reaction (RT-PCR) was positive for human T-lymphotropic virus type 1 (HTLV-1), and the patient was diagnosed with stage IV ATLL given ascitic fluid involvement. 

Hospital course

The patient’s hospital course was complicated by spontaneous tumor lysis syndrome, recurrent malignant small bowel obstruction, and bacteremia. Therapeutic large-volume paracentesis and thoracentesis were performed for persistent abdominal swelling, tachypnea, and a large loculated pleural effusion. Plans were made to initiate etoposide phosphate, vincristine sulfate (Oncovin), cyclophosphamide, and doxorubicin hydrochloride (hydroxydaunomycin) (EPOCH) chemotherapy for stage IV ATLL, and a port was placed for treatment.

During the port placement, the patient experienced a large-volume emesis aspiration while under anesthesia, which required emergent intubation. In the ICU, the patient experienced difficulty oxygenating due to recurrent mucus plugging, which resulted in high peak pressures and ultimately led to bradycardic cardiac arrest. Return of spontaneous circulation (ROSC) was achieved after 10 minutes, but the patient sustained anoxic brain injury. Unfortunately, the patient passed away 32 days after admission.

Outcome

This case highlights the aggressive nature of stage IV ATLL with peritoneal involvement, an uncommon clinical manifestation. Despite intensive diagnostic efforts and multidisciplinary management, the patient’s course was marked by severe complications and a poor outcome, underscoring the challenges associated with this rare disease.

## Discussion

Peritoneal lymphomatosis is a rare complication of lymphomas that often mimics peritoneal carcinomatosis on imaging [3]. It is most commonly associated with high-grade B-cell lymphomas, including diffuse large B-cell lymphoma and Burkitt lymphoma [4-8]. T-cell lymphomas, including ATLL, are rare, with only a few thousand cases documented worldwide annually [9]. Between 2001 and 2015, 2,148 ATLL cases were identified in the United States, with 18% occurring in New York State [10].

ATLL is an aggressive and uncommon mature peripheral T-cell lymphoma caused by HTLV-1 [11]. In the United States, areas with significant immigrant populations from endemic regions have an ATLL incidence recorded at 3.2 per 100,000 individuals [12]. While ATLL in individuals of Caribbean descent is less extensively studied compared to those of Japanese descent, research from immigrant populations in New York State highlights the urgent need for targeted early treatment due to the disease's poor response to chemotherapy and unfavorable prognosis [13,14].

In our patient, the initial diagnostic workup was focused on peritoneal carcinomatosis, commonly associated with gastrointestinal cancers. However, ATLL was diagnosed only after an omental biopsy revealed atypical lymphoid cells with a Ki-67 proliferative index of 90%. Immunohistochemical analysis showed positivity for CD3, CD2, and CD4, with loss of CD5 and CD7. Serological tests confirmed HTLV-1 positivity, establishing the diagnosis of ATLL. Consistent with the findings in prior literature, the patient experienced a highly aggressive clinical course.

A study examining pathologic specimens from 34 patients provides one of the few references to peritoneal involvement in ATLL [15]. This study identified two cases of peritoneal effusions that tested positive for atypical lymphoid cells with cytometric features of ATLL, in addition to seven cases of pleural effusions [15]. Additionally, a 1995 case report described a 62-year-old Japanese man with ATLL, presenting without lymphadenopathy but with positive cytology for ATLL in ascitic and pleural fluid [16]. Another longitudinal cohort study, conducted over 14 years and involving 60 patients with ATLL from Caribbean and Latin American populations, found that lymphadenopathy was the most common presentation among these patients [17]. Of these 60 patients, 23 had extranodal involvement, including 13 with skin involvement and six with pulmonary involvement [17]. Notably, there were no cases of peritoneal effusions or pleural involvement in this cohort. Additionally, 65% of the patients exhibited hypercalcemia and leukocytosis, which was absent in our patient.

The potential for peritoneal involvement in ATLL highlights the importance of using 18F-FDG PET-CT, rather than CT alone, for staging, as PET-CT has greater sensitivity for detecting lymphoma at extranodal sites [18]. In a retrospective series of 12 patients (11 with non-Hodgkins and one with Hodgkin's lymphoma), the most frequently observed imaging features of lymphomatous involvement of the peritoneum were peritoneal thickening and nodularity. Even when the thickening was subtle or barely detectable, diffuse FDG uptake along the peritoneal surfaces was considered a strong indicator of peritoneal involvement [19]. Additionally, two case reports. that is, one of a patient with Burkitt’s lymphoma and the other with diffuse large B-cell lymphoma, which initially showed intense peritoneal FDG uptake ("peritoneal super scan"), later resolved on PET-CT, indicating a complete metabolic response and highlighting the possible useful role of PET-CT in disease monitoring [20].

Ultimately, the diagnosis of peritoneal lymphomatosis depends on biopsy, with ultrasound or CT-guided biopsy emerging as a safe and effective method for obtaining these samples [4,21]. Flow cytometry, revealing the expression of pan-T-cell markers and CD25 on neoplastic cells, along with HTLV-1 serology, is critical for establishing the diagnosis of ATLL [22].

## Conclusions

This case emphasizes the need to consider endemic contexts when pursuing a diagnosis in peritoneal carcinomatosis of unknown origin. It also underscores the utility of PET-CT in guiding prompt biopsy when evaluating peritoneal malignancies, thereby facilitating timely diagnosis and management of this aggressive condition. Further studies tracking the sensitivity and specificity of serial PET/CTs for disease tracking could provide valuable insights into optimizing monitoring strategies and improving outcomes for patients with peritoneal lymphomatosis.
